# Honokiol Inhibits Arsenic Trioxide–Induced Cardiomyopathy by Modulating Ferroptosis via SIRT3 Signaling Pathway

**DOI:** 10.1155/crp/8878178

**Published:** 2026-08-02

**Authors:** Qing-qing Wei, An-Liang Huang, Ning-yi Liu, Yi-ran Zhang, Fan Yang

**Affiliations:** ^1^ School of Basic Medicine, North Sichuan Medical College, Nanchong, Sichuan, China, scu.edu.cn; ^2^ Department of Pathology, Chengdu Fifth People’s Hospital The Second Clinical Medical College, Cancer Prevention and Treatment Institute of Chengdu, Affiliated Fifth People’s Hospital of Chengdu University of Traditional Chinese Medicine, Chengdu, Sichuan, China, scu.edu.cn; ^3^ School of Medical and Life Sciences, Chengdu University of Traditional Chinese Medicine, Chengdu, Sichuan, China, scu.edu.cn

**Keywords:** arsenic trioxide, autophagic flux, cardiotoxicity, ferroptosis, Honokiol, SIRT3

## Abstract

Ferroptosis has recently been identified as a critical mechanism underlying arsenic trioxide (ATO)–induced cardiotoxicity. This study aimed to determine whether Honokiol (HKL) protects against ATO‐induced cardiac injury by inhibiting ferroptosis and to investigate the role of the SIRT3 signaling pathway in this process. Using 129S1/SvImJ wild‐type (WT) and SIRT3‐knockout (SIRT3^−/−^) mice, we demonstrated that HKL attenuates ATO‐induced myocardial injury and hypertrophy in a SIRT3‐dependent manner. Mechanistically, HKL reduced oxidative stress through the SIRT3/SOD2 pathway, as evidenced by decreased mitochondrial ROS production and SOD2 acetylation, along with preserved mitochondrial ATP generation capacity. Furthermore, HKL inhibited ferroptosis, indicated by reduced iron content, MDA levels, and 4‐HNE expression, along with restored GPX4 and GSH levels. Notably, HKL restored autophagic flux impaired by ATO, and this effect was associated with its antiferroptotic and cardioprotective actions. Pharmacological or genetic inhibition of SIRT3 or autophagic flux abolished the protective effects of HKL. Autophagic flux is closely associated with the protective effect of Honokiol on ATO‐induced ferroptosis. These findings reveal that HKL protects against ATO‐induced cardiomyopathy by restoring autophagic flux and inhibiting ferroptosis via the SIRT3 signaling pathway.

## 1. Introduction

Arsenic trioxide contributes to cardiovascular toxicity by disrupting mitochondrial function through multiple mechanisms, particularly by promoting the opening of mitochondrial permeability transition pores and damaging components of the electron transport system [1]. Mitochondrial dysfunction leads to increased reactive oxygen species (ROS) production and the release of proapoptotic factors, promoting both apoptotic and ferroptotic cell death pathways [2, 3]. Ferroptosis is triggered by dysregulation of cellular iron metabolism, glutathione depletion, and inactivation of key antioxidant systems such as glutathione peroxidase 4 (GPX4) and the cystine/glutamate antiporter (system xc^−^). This process is characterized by accumulation of ROS and lipid peroxides, leading to cellular damage and death [4]. ROS overproduction and ferroptosis can create a vicious cycle, as ROS initiate lipid peroxidation, which in turn generates more ROS, intensifying oxidative stress. Therefore, regulation of ROS production by enzymatic and nonenzymatic antioxidant systems is essential for cellular homeostasis. Studies have demonstrated that ROS overproduction in cardiomyocytes is the primary mechanism of ATO‐induced cardiotoxicity [5]. Although antioxidants may partially protect cardiomyocytes from oxidative damage, the clinical efficacy of antioxidant monotherapy against ATO cardiotoxicity remains limited [6]. Thus, exploring novel therapeutic strategies to mitigate ATO‐induced cytotoxicity is a major challenge.

Autophagy is a self‐degradative process that removes misfolded proteins and damaged organelles, playing an essential role in maintaining cellular homeostasis. While autophagy was once considered a cell death mechanism, it is now recognized as a critical protective process [7]. Impaired autophagy in cardiomyocytes, particularly disruption of autophagic flux, contributes to the pathogenesis of heart failure, hypertrophic cardiomyopathy, dilated cardiomyopathy, cardiac aging, diabetic cardiomyopathy, and ischemia/reperfusion injury [8]. Autophagic flux helps mitigate oxidative stress by clearing damaged cellular components, thereby indirectly influencing the initiation of ferroptosis [9]. In the context of ATO‐induced cardiotoxicity, studies have shown that in cardiomyocytes treated with 6 μM ATO, elimination of dysfunctional mitochondria via the Parkin pathway may be impaired due to inhibition of mitophagy [6]. Additionally, ATO induces ROCK‐dependent apoptosis in H9c2 cells, and ROCK inhibition with Y‐27632 not only suppresses apoptosis but also accelerates autophagy, which is necessary for protection against ATO toxicity [10]. Blocked autophagic flux fails to clear damaged organelles and proteins caused by oxidative stress, thereby exacerbating oxidative damage [11]. Therefore, restoring autophagic flux may represent a novel therapeutic strategy to reduce ATO cardiotoxicity. However, effective approaches to target autophagic flux in this context remain poorly explored.

Honokiol, a bioactive compound derived from Magnolia bark, exhibits diverse pharmacological properties. In oncology, HKL demonstrates potent anticancer effects [12]. In neurological disorders, HKL shows neuroprotective effects in neurodegenerative diseases such as Alzheimer’s and Parkinson’s [13]. In metabolic and infectious diseases, HKL exhibits antiobesity and antimicrobial activities by influencing lipid metabolism, reducing adipogenesis, improving insulin sensitivity, disrupting microbial membranes, and inhibiting pathogen growth [14, 15]. Regarding cardiovascular protection, HKL acts as a potent antioxidant, scavenging ROS and enhancing endogenous antioxidant enzyme expression [16]. Moreover, HKL prevents cardiomyocyte apoptosis by modulating both intrinsic and extrinsic apoptotic pathways, increasing antiapoptotic proteins (e.g., Bcl‐2) and reducing proapoptotic factors (e.g., Bax and caspases) [17]. Additionally, HKL stabilizes mitochondrial membranes, reduces mitochondrial ROS production, and preserves ATP levels, ensuring proper mitochondrial function under stress conditions [14, 16–18]. In ischemia–reperfusion injury models, HKL reduces infarct size and improves postinjury cardiac function [17]. Our previous study demonstrated that ATO causes cardiomyocyte dysfunction in mice and cultured cardiomyocytes, and HKL exerts protective effects against ATO‐induced myocardial toxicity [19]. However, the detailed mechanism underlying this protection remains incompletely understood. A recent study showed that the ferroptosis inhibitor Fer‐1 protects H9c2 cells from ATO‐induced apoptosis, suggesting that inhibiting ferroptosis could alleviate ATO cardiotoxicity [20]. Whether HKL protects against ATO‐induced cardiotoxicity by inhibiting ferroptosis has not been investigated.

Autophagy activation by HKL has been reported to protect against various forms of cardiac injury, including ischemia–reperfusion injury [21] and autoantibody‐induced cardiotoxicity [22]. By enhancing clearance of damaged components and maintaining cellular homeostasis, HKL‐mediated autophagy may contribute to cardiomyocyte survival and functional recovery. Considering the involvement of redox reactions and sirtuins in regulating autophagy and oxidative stress [23], we hypothesized that HKL may improve autophagic flux to prevent ATO cardiotoxicity. The present study was designed to examine whether HKL improves autophagic flux and modulates ferroptosis via the SIRT3 pathway, thereby protecting cardiomyocytes against ATO‐induced injury. This study is the first to demonstrate that HKL alleviates ATO‐induced cardiomyopathy by restoring autophagic flux and inhibiting ferroptosis in a SIRT3‐dependent manner, providing a novel mechanistic framework for its cardioprotective effects.

## 2. Materials and Methods

### 2.1. Reagents

Honokiol with purity > 98% (Sigma‐Aldrich, St. Louis, MO, USA) was dissolved in corn oil. Arsenic trioxide, 3‐TYP, and bafilomycin A1 were obtained from Sigma‐Aldrich. ATO was dissolved in 1.65 M NaOH at 5 × 10^−2^ M as a stock solution. Monoclonal antibodies against GAPDH (Cat. #2118), p62 (Cat. #23214), and SIRT3 (Cat. #5490) were purchased from Cell Signaling Technology (Beverly, MA, USA). Antibodies against GPX4 (ab125066), 4‐HNE (ab48506), Ac‐SOD2 (acetyl K122, ab214675), and SOD2 (ab68155) were obtained from Abcam (Cambridgeshire, UK). The protein assay kit was purchased from Bio‐Rad Laboratories (Hercules, CA, USA). ATP bioluminescence assay kit was obtained from Beyotime Biotechnology (Shanghai, China). All chemicals used were of analytical grade and culture grade. The doses of Honokiol (0.2 mg/kg for in vivo, 10 μM for in vitro) were selected based on our previous study [19] and preliminary experiments showing optimal cardioprotective effects without cytotoxicity.

### 2.2. Adult Mouse Cardiomyocyte Isolation and Culture

Primary cultures of cardiomyocytes were prepared from adult mouse hearts as previously described [19]. Ethical approval was provided by the Ethics Committee of State Key Laboratory of Biotherapy, Sichuan University. Briefly, adult C57BL/6 male mice (8–10 weeks old) were anesthetized with isoflurane. After chest opening, the heart was immediately flushed by an injection of 7 mL of EDTA buffer into the left ventricle and then transferred to a fresh dish. EDTA buffer, perfusion buffer, and collagenase buffer were consecutively injected into the left ventricle to digest the heart tissue. The heart was gently pulled into pieces using sterile forceps and pipetted until most myocytes were completely dissociated. Stop buffer was then added. The cell suspension was filtered through a 100 μm nylon mesh and placed in a conical tube upright to allow cells to settle by gravity. Cells underwent four sequential rounds of gravity settling using three intermediate calcium buffers to gradually restore calcium concentration to physiological levels. After removing the supernatant in the final round, cells were collected and plated in laminin‐coated culture plates. The majority of cells exhibited rod‐shaped morphology with crisp striations. Twenty‐four hours after plating, cardiomyocyte cultures were treated with corn oil, HKL (10 μM), ATO (4 μM), or HKL (6 h before ATO) + ATO. For SIRT3 inhibition, 3‐TYP (1 μM) was added 6 h before HKL treatment followed by ATO administration. Twenty‐four hours after treatment, cells were harvested for ROS determination, apoptosis assays, caspase‐3 activation assays, and western blot analysis. To assess autophagic flux, cardiomyocytes were treated with bafilomycin A1 (10 nM) and ATO (4 μM) in the presence or absence of HKL (10 μM) for 12 h.

### 2.3. Animal Study

129S1/SvImJ male wild‐type mice (Strain #002448) and SIRT3‐knockout mice (Strain #012755) with 129S6 background, 8–10 weeks old, weighing 22–25 g, were obtained from Jackson Laboratory (Bar Harbor, ME, USA). Ethical approval was provided by the Ethics Committee of State Key Laboratory of Biotherapy, Sichuan University (approval no: 20230302090). Mice were maintained in a specific pathogen‐free facility with 12‐h light–dark cycles. All animals were randomly assigned to experimental groups. Echocardiography and histological analyses were performed by investigators blinded to group allocation. All methods are reported in accordance with ARRIVE guidelines (https://arriveguidelines.org). Forty‐eight mice were randomly assigned into eight groups (*n* = 6 per group): (1) vehicle group; (2) SIRT3‐KO + vehicle group; (3) ATO group; (4) SIRT3‐KO + ATO; (5) ATO + HKL group; (6) SIRT3‐KO + ATO + HKL group; (7) CQ + vehicle group; and (8) CQ + ATO + HKL group. Mice with or without SIRT3 knockout were injected intraperitoneally (i.p.) with ATO at a dose of 4 mg/kg once every 4 days for a total of 4 injections. HKL treatment (0.2 mg/kg, i.p.) started 6 h before the first ATO treatment. In control mice, vehicle (corn oil) was used [19]. To block autophagic flux, chloroquine (50 mg/kg, i.p.) was administered to mice daily. After echocardiographic assessment (3 days after the last administration), all animals were euthanized by intraperitoneal injection of sodium pentobarbital (150 mg/kg body weight) and sacrificed, and hearts were collected and fixed with formalin for histological analysis. Baseline echocardiographic parameters and mitochondrial function in SIRT3‐KO mice were comparable to those in wild‐type mice, indicating that SIRT3 deficiency alone does not induce overt cardiac dysfunction under basal conditions (Table 1).

**TABLE 1:** Echocardiographic analysis: data are expressed as means ± SD (*n* = 6 per group).

Groups	Ejection fraction (%)	Fractional shortening (%)	LVIDD (mm)	LVIDS (mm)	Heart rate (systolic, bpm)
Vehicle	85.22 ± 2.84	55.23 ± 1.81	3.62 ± 0.17	1.77 ± 0.15	448.42 ± 39
SIRT3‐KO + vehicle	85.12 ± 3.08	55.72 ± 2.12	3.61 ± 0.27	1.76 ± 0.22	457.74 ± 43
ATO	51.79 ± 4.93^∗^	27.89 ± 4.23^∗^	4.02 ± 0.28^∗^	3.15 ± 0.17^∗^	482.49 ± 47
SIRT3‐KO + ATO	49.37 ± 5.79^∗^	24.46 ± 4.21^∗^	4.48 ± 0.39^∗^	3.34 ± 0.28^∗^	487.25 ± 49
ATO + HKL	81.37 ± 3.21^#^	47.81 ± 3.78^#^	3.15 ± 0.17^#^	1.57 ± 0.18^#^	479.74 ± 42
SIRT3‐KO + HKL + ATO	54.27 ± 5.61^&^	30.18 ± 4.51^&^	3.96 ± 0.23^&^	2.89 ± 0.21^&^	464.74 ± 40

*Note:* Echocardiographic parameters were obtained from each mouse, and the values from six mice per group were used for statistical analysis.

Abbreviations: LVIDD, left ventricular internal diameter diastole; LVIDS, left ventricular internal diameter systole.

^∗^ *p* < 0.05 vs. vehicle.

^#^ *p* < 0.05 vs. ATO.

^&^ *p* < 0.05 vs. ATO + HKL (two‐way ANOVA with Tukey’s post hoc test).

### 2.4. Isolation of Mitochondria From Mouse Heart

Mitochondria were prepared from adult mouse hearts as previously described [19]. Freshly harvested hearts were dissected and dissociated into small pieces (∼200 mg) using surgical scissors. After clearing blood vessels, tissues were homogenized with a Dounce homogenizer in chilled isolation buffer (225 mM D‐mannitol, 75 mM sucrose, 5 mM KH_2_PO_4_, and 0.5 mM EDTA, pH 7.4) in an ice‐cold bath. The homogenate was centrifuged at 200 × g for 5 min at 4°C. After collecting the supernatant, the pellet was resuspended in isolation buffer and recentrifuged at 1000 × g for 10 min at 4°C. The resulting supernatant containing mitochondria was centrifuged at 8000 × g for 10 min at 4°C. The final mitochondrial pellet was resuspended in chilled respiration‐compatible buffer (225 mM D‐mannitol, 75 mM sucrose, 5 mM KH_2_PO_4_, and 10 mM Tris–HCl, pH 7.0). Mitochondrial purity and integrity were assessed by lactate dehydrogenase and succinate dehydrogenase assays.

### 2.5. Assessment of Oxidative Stress

The oxidation‐sensitive fluorescent probe 5‐(and‐6)‐carboxy‐2′,7′‐dichlorodihydrofluorescein diacetate (carboxy‐DCFDA) was used to measure the redox state of cells and heart tissue lysates as previously described [19]. Cells or heart tissue lysates were incubated with carboxy‐H_2_DCFDA at a final concentration of 1 μM in regular culture medium for 30 min in the dark and washed twice with PBS. Fluorescence density (arbitrary units, AU) was acquired using a fluorescence plate reader. To detect hydrogen peroxide levels, freshly isolated mitochondria from heart tissues were incubated with Amplex Red reagent (10‐acetyl‐3,7‐dihydroxyphenoxazine) according to the manufacturer’s instructions (Amplex Red hydrogen peroxide/peroxidase assay kit, ThermoFisher Scientific) as previously described [19]. Briefly, 50 μL of sample from each group, a positive control (10 μM diluted 20 mM H_2_O_2_ working solution in 1 × reaction buffer), and a negative control (1 × reaction buffer without H_2_O_2_) were loaded into individual wells of a microplate. Fifty microliters of working solution containing 100 μM Amplex Red reagent and 0.2 U/mL of HRP were added to each well. The microplate was incubated at room temperature for 30 min (protected from light), and absorbance was measured at 560 nm in a 96‐well microplate reader.

To measure mitochondrial superoxide, the fluorescent probe MitoSOX Red was used according to the manufacturer’s protocol. Images were captured using a Leica TCS SPE confocal microscope. Mean whole‐cell fluorescence of MitoSOX Red was quantified using ImageJ software. Malondialdehyde (MDA) content and glutathione (GSH) concentration were measured in cell lysates using commercial assay kits according to the manufacturer’s instructions (Cayman Chemical Company, USA).

### 2.6. Assessment of Mitochondrial ATP Production

To assess mitochondrial function, ATP levels were measured in freshly isolated cardiac mitochondria and whole heart tissue lysates. Mitochondria were isolated as described above. For mitochondrial ATP production measurement, freshly isolated mitochondria (50 μg protein) were resuspended in respiration buffer (125 mM KCl, 10 mM Tris‐MOPS, 5 mM glutamate, 2.5 mM malate, 2 mM KH_2_PO_4_, pH 7.4) and incubated with 0.5 mM ADP at 37°C for 10 min. ATP content was determined using a luciferin‐luciferase‐based ATP bioluminescence assay kit (Beyotime, China) according to the manufacturer’s protocol. Luminescence was measured using a microplate reader (SpectraMax iD3, Molecular Devices). Results were normalized to mitochondrial protein concentration and expressed as nmol ATP/mg protein. For tissue ATP content, frozen heart tissues were homogenized in ice‐cold ATP lysis buffer, and ATP levels were measured using the same kit, normalized to tissue weight.

### 2.7. Iron Content Measurement

Heart tissues were homogenized, and iron content was determined using a commercially available kit (Nanjing Jiancheng, China) following the manufacturer’s instructions. Briefly, fresh heart tissues were homogenized in iron assay buffer on ice and centrifuged at 4°C. Iron reducing agent was added, and the supernatant was collected after 30 min at 37°C. Iron probe was then added, and the reaction continued for 60 min at the same temperature. Absorbance was measured at 593 nm using a fluorescence plate reader.

### 2.8. Cell Death Assay

ATO‐induced cardiac cell death was assessed using Hoechst 33342/Annexin V staining as previously described [19]. Cardiomyocytes were cultured in 6‐well plates for 24 h. After designated treatments, cells were washed with Annexin V binding buffer, incubated with Annexin V‐FITC for 15 min at room temperature, and counterstained with Hoechst 33,342 for nuclei. After washing twice with PBS, images of dead cells were captured using fluorescence microscopy. Although Annexin V staining and LDH release are not exclusive to ferroptosis, the use of the specific ferroptosis inhibitor Fer‐1 prior to ATO exposure significantly reduced cell death, supporting the involvement of ferroptosis in ATO‐induced cardiomyocyte injury.

### 2.9. Western Blot Analysis

Briefly, 1 × 10^5^ primary cultured cardiomyocytes were lysed in lysis buffer. Frozen cardiac tissues were lysed in RIPA buffer. Cells were centrifuged at 12,500 rpm for 30 min. Protein concentration of the supernatant was determined using the Bio‐Rad protein assay kit. Whole‐cell lysates after denaturation were separated by 10% SDS‐PAGE. Gels were electroblotted onto polyvinylidene difluoride membranes. Membrane blots were blocked at 4°C in 5% nonfat dry milk overnight and incubated with each antibody at recommended dilution for 8 h at 37°C. After rinsing in solution containing 10 mM Tris‐HCl pH 7.5, 100 mM NaCl, and 0.1% Tween‐20 (TBS‐T), membranes were incubated with horseradish peroxidase–conjugated secondary antibodies at a dilution of 1:10,000. Immunoreactive bands were detected by enhanced chemiluminescence (Amersham Corp., Arlington Heights, IL, USA) followed by autoradiography. Equal loading was confirmed by detection of GAPDH.

### 2.10. Echocardiographic Assessment

Echocardiography was performed in anesthetized mice (ketamine 100 mg kg^−1^ and xylazine 5 mg kg^−1^, i.p.) using a Vevo 2100 Imaging System (VisualSonics, Inc.) equipped with a 15 MHz linear transducer as previously described [19]. B‐mode was used to obtain the ventricular section at the level of the mitral papillary muscle. Systolic and diastolic ventricular wall thickness, ventricular diameter, and ventricular wall movement were monitored in M‐mode. The percentage of fractional shortening (FS%), ejection fraction (EF%), and other parameters were obtained automatically. Echocardiographic parameters were measured once per mouse, and the mean values of six mice per group were used for statistical analysis.

### 2.11. Histological Analysis

After excision and washing with PBS, hearts were fixed in 10% formalin and embedded in paraffin according to standard histological procedures. Hearts were sectioned at 4‐μm thickness. Sections were stained with phycoerythrin‐conjugated WGA (wheat germ agglutinin, Invitrogen) to determine myocyte cross‐sectional area and stained for GPX4 and 4‐HNE to assess ferroptosis. Myocardial SIRT3 and acetylated‐SOD2 expression were also assessed by immunohistochemistry. Images were captured by microscopy, and individual cell size was measured using ImageJ software. More than 100 myocytes from sections of at least six different mouse samples were analyzed per group. IHC staining images were observed and captured using an automatic digital slice scanning system (VS200, OLYMPUS, Japan) and processed with ImageJ software. All figures should be provided in a high‐resolution (≥ 300 dpi) format with consistent labeling and scale bars.

### 2.12. Statistical Analysis

All quantitative data are expressed as means ± SD. All animals were randomly assigned to experimental groups. Echocardiography and histological analyses were performed by investigators blinded to group allocation. Statistical comparisons between more than two groups were performed using two‐way analysis of variance (ANOVA) followed by Tukey’s post hoc test. *p* values < 0.05 were defined as statistically significant.

## 3. Results

### 3.1. SIRT3 Knockout Abolishes Protective Effects of HKL in ATO‐Induced Myocardial Injury and Hypertrophy

**FIGURE 1:**
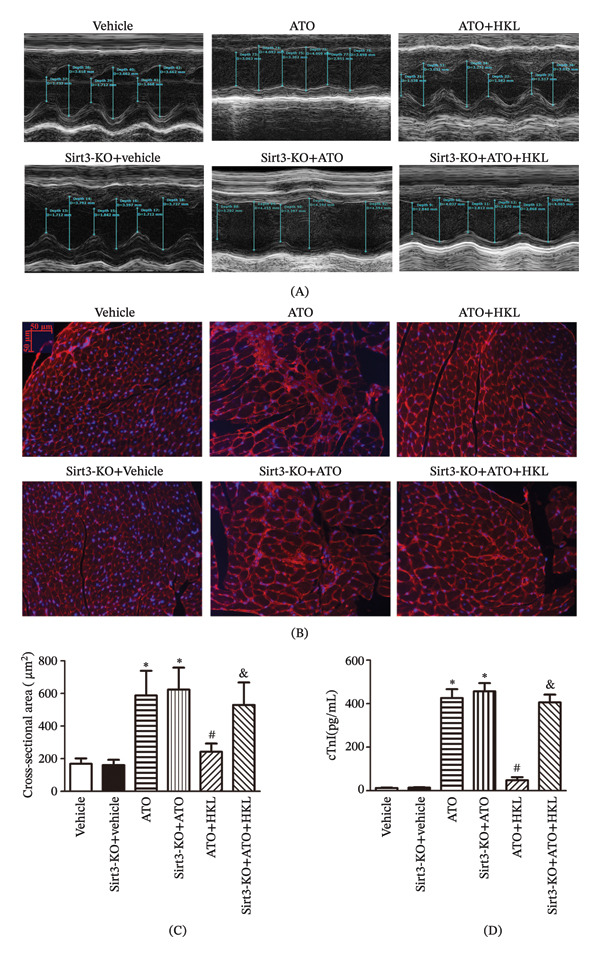
SIRT3 knockout abolishes protective effects of HKL in ATO‐induced myocardial injury and hypertrophy. C57BL/6 WT and SIRT3‐KO mice were injected with HKL (0.2 mg/kg, i.p.) 6 h before ATO treatment (4 mg/kg/day, i.p., once every 4 days for a total of 4 injections). 24 h after the final ATO treatment, echocardiography was performed in anesthetized mice to monitor changes in cardiac function (A). Representative images show WGA (red) and Hoechst 33,342 (blue) staining of formalin‐fixed, paraffin‐embedded left ventricular myocardial tissue sections (B), and statistical analysis of cardiomyocyte cross‐sectional area (C). Serum troponin I levels were measured (D). Data are expressed as means ± SD, *n* = 6–8. ^∗^ *p* < 0.05 vs. vehicle; ^#^ *p* < 0.05 vs. ATO‐exposed mice; ^&^ *p* < 0.05 vs. HKL + ATO‐exposed mice (two‐way ANOVA with Tukey’s post hoc test).

To investigate whether SIRT3 knockout reduces the protective effect of HKL against ATO‐induced cardiotoxicity in vivo, the effects of HKL on ATO exposure were examined in both wild‐type and SIRT3‐KO mice. As shown in Figure 1A and Table 1, SIRT3‐KO diminished the HKL‐pretreatment‐induced improvement in ejection fraction (EF%) and fractional shortening (FS%) in ATO‐treated wild‐type mice. Other echocardiographic parameters are shown in Table 1. Furthermore, we investigated whether the regulatory effect of HKL on the SIRT3 activity is required for its antihypertrophic effect. WGA staining demonstrated that SIRT3‐KO almost completely blocked the protective effects of HKL against ATO‐induced cardiac hypertrophy (Figure 1B,C). Consistent with echocardiographic findings, ATO exposure led to a significant increase in serum troponin I, and HKL pretreatment effectively reduced serum cTnI; however, this effect was neutralized by SIRT3 knockout (Figure 1D).

Honokiol protects myocardium from ATO‐induced oxidative stress through the SIRT3/SOD2 pathway.

**FIGURE 2:**
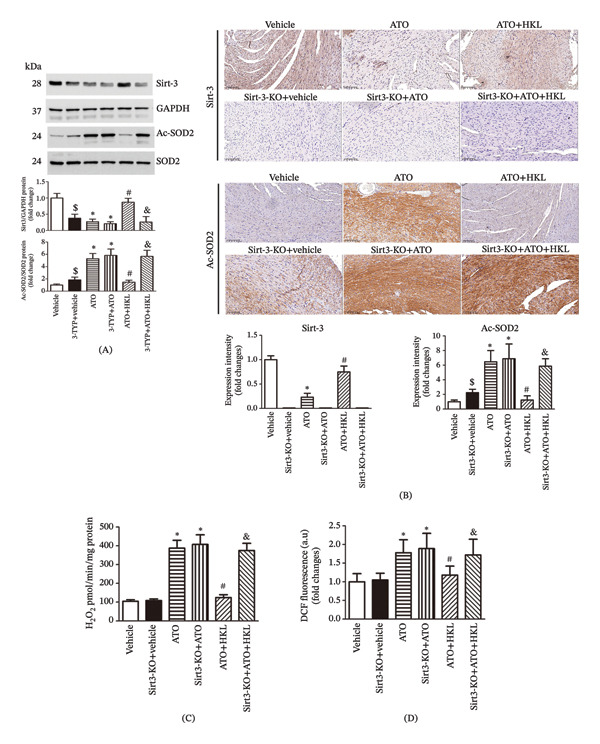
Honokiol protects cardiomyocytes from ATO‐induced oxidative stress through the SIRT3/SOD2 pathway. Primary cultured cardiomyocytes from each group were analyzed by western blot (A). Myocardial tissue sections of left ventricular specimens were analyzed by immunohistochemistry for the indicated antibodies (magnification = 200×) (B). Myocardial Ac‐SOD2 was significantly increased, while SIRT3 expression was markedly downregulated by ATO treatment, which was reversed by HKL pretreatment. SIRT3 knockout nullified the protective effect of HKL on SOD2 acetylation. ROS (H_2_O_2_) production in isolated heart mitochondria was measured by amplex red (C). ROS generation in whole tissue lysates of hearts from each group was measured by DCFDA using a fluorescence plate reader (D). SIRT3 knockout largely abolished the antioxidant properties of HKL following ATO exposure. Data are expressed as means ± SD, *n* = 3–8. ^∗^ *p* < 0.05 vs. vehicle; ^#^ *p* < 0.05 vs. vehicle + ATO; ^&^ *p* < 0.05 vs. ATO + HKL (two‐way ANOVA with Tukey’s post hoc test).

Our previous work showed that HKL effectively reduces ROS upregulation in heart tissues exposed to ATO. To determine the mechanism by which HKL reduces oxidative stress, we examined SIRT3 and Ac‐SOD2 (acetylated SOD2) levels in primary cultured cardiomyocytes and heart tissues by western blot and IHC, respectively. In both in vitro and in vivo models, compared with the sham group, SIRT3 expression in the ATO group was significantly decreased, while SOD2 acetylation was significantly increased. Compared with the ATO group, HKL significantly upregulated SIRT3 expression and decreased SOD2 acetylation (Figure 2A,B). To determine whether the antioxidant effects of HKL were mediated via SIRT3 activation, 3‐TYP or SIRT3‐KO mice were used. As shown in Figure 2C,D, SIRT3 knockout largely abolished the antioxidant properties of HKL following ATO exposure, as evidenced by the increased mitochondrial ROS (H_2_O_2_) generation (Amplex Red) and total oxidative stress levels (DCF fluorescence) in heart tissues. Similarly, SIRT3 knockout also offset the decreased Ac‐SOD2 expression compared with that in the wild‐type group (Figure 2A,B).

Consistent with the increased oxidative stress, mitochondrial dysfunction was evidenced by a significant decline in both mitochondrial ATP production capacity and total tissue ATP content in the ATO‐treated group compared to that of the vehicle group (Supplemental Figure 1A, 1B). Honokiol pretreatment preserved mitochondrial ATP generation, an effect that was abrogated in SIRT3‐KO mice, suggesting that SIRT3 contributes to maintaining mitochondrial bioenergetics under ATO stress.

### 3.2. Ferrostatin‐1 Decreases Cardiomyocyte Apoptosis Induced by ATO

**FIGURE 3:**
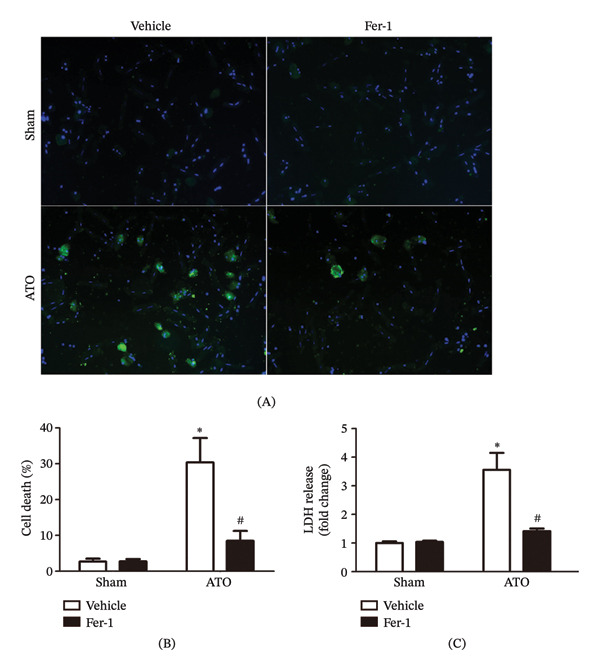
Effects of ferrostatin‐1 on ATO‐induced apoptosis in primary cultured cardiomyocytes. Cardiomyocytes were pretreated with ferrostatin‐1 (40 μM) for 12 h followed by sham or 4 μM ATO treatment. Inhibition of ATO‐induced cell death was assessed through Hoechst 33,342 (blue)/annexin V (green) staining. Cell images were captured under fluorescence and UV light microscopy; blue spots represent cell nuclei, and green spots represent apoptotic bodies (A). The rate of cell death was counted under microscopy (B). LDH leakage activity was measured using a colorimetric lactate dehydrogenase assay kit (C). Data are expressed as means ± SD from three independent experiments. ^∗^ *p* < 0.05 vs. vehicle in sham; ^#^ *p* < 0.05 vs. vehicle in ATO‐treated cells (two‐way ANOVA with Tukey’s post hoc test).

To determine whether ferroptosis is involved in ATO‐induced cardiomyopathy, primary cultured cardiomyocytes were pretreated with Ferrostatin‐1 (40 μM) for 12 h and then treated with 4 μM ATO for 24 h. Cell death was analyzed by Hoechst 33342/Annexin V staining and LDH leakage assay. As shown in Figure 3, the number of Annexin V‐positive cells increased after ATO exposure; in contrast, in the presence of Ferrostatin‐1, ATO‐induced cardiac cell death was significantly attenuated (Figure 3A,B). LDH activity was detected using a colorimetric lactate dehydrogenase assay kit. Our results (Figure 3C) show that LDH activity was significantly increased in primary cultured cardiomyocytes 24 h after exposure to 4 μM ATO, whereas Ferrostatin‐1 pretreatment significantly suppressed ATO‐induced LDH leakage.

### 3.3. Honokiol Inhibits ATO‐Induced Ferroptosis via the SIRT3 Pathway in Myocardial Tissues

**FIGURE 4:**
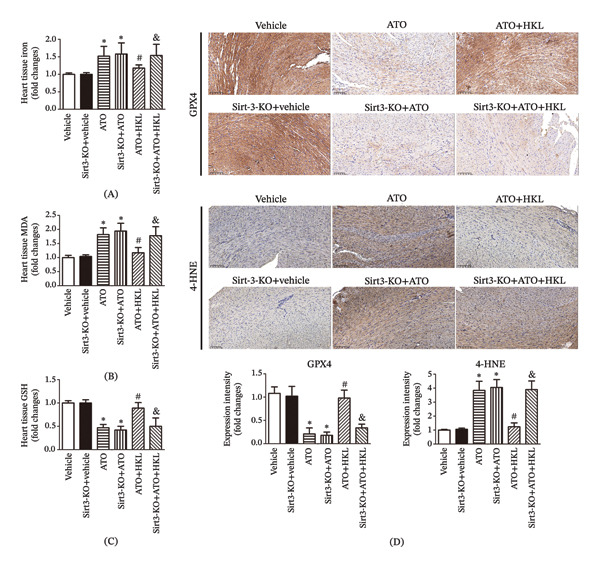
Effects of Honokiol on ferroptosis‐related biomarkers in heart tissues of ATO‐treated mice. Three days after the last administration, animals were sacrificed, and hearts were collected. Iron content (A), MDA levels (B), and GSH levels (C) of heart tissues were measured. Heart tissues were embedded in paraffin and sectioned at 4‐μm thickness. GPX4 and 4‐HNE expressions were analyzed by immunohistochemistry (magnification = 200×) (D). Data are expressed as means ± SD, *n* = 6–8. ^∗^ *p* < 0.05 vs. vehicle; ^#^ *p* < 0.05 vs. vehicle + ATO; ^&^ *p* < 0.05 vs. ATO + HKL.

Ferroptosis has been shown to be a programmed form of cardiomyocyte death caused by ATO [20]. In the present study, ATO‐treated mice showed increased cardiac tissue iron content and MDA levels, accompanied by decreased GSH levels (Figure 4A–C). In addition, IHC analysis showed reduced GPX4 expression and increased 4‐HNE levels in cardiac tissues of ATO‐treated mice (Figure 4D). Pretreatment with HKL reduced iron overload in ATO‐treated mice, reversed the ATO‐induced increase in cardiac MDA, and attenuated the decrease in GSH. Moreover, HKL pretreatment reversed the decline in GPX4 expression and inhibited 4‐HNE expression in myocardial tissues of ATO‐treated mice. However, in SIRT3‐KO mice, HKL pretreatment failed to reverse these ferroptosis‐associated markers in myocardial tissues exposed to ATO. These data indicate that HKL ameliorates ATO‐induced myocardial ferroptosis via the SIRT3 signaling pathway.

### 3.4. Honokiol Improves Autophagic Flux in Cardiomyocytes Exposed to ATO

**FIGURE 5:**
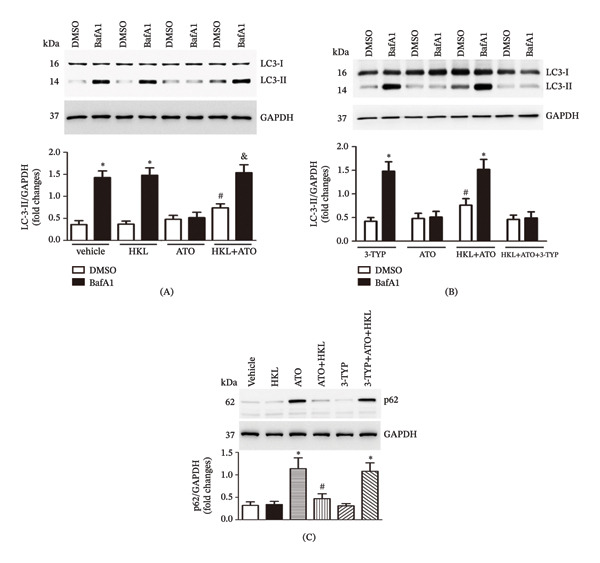
Honokiol improves ATO‐induced diminishment of autophagic flux in cardiomyocytes. Cultured cardiomyocytes were pretreated with HKL for 6 h followed by sham or 4 μM ATO treatment for 24 h, then bafilomycin A1 (BafA1) was added for another 2 h. Lysates from primary cultured cardiomyocytes were collected at the end of treatment. LC3‐II and GAPDH expression levels were detected by western blot. A representative western blot is shown in the upper panel, with corresponding statistical graph in the lower panel (A). Cultured cardiomyocytes were pretreated with HKL for 6 h followed by 4 μM ATO treatment with or without SIRT3 inhibitor 3‐TYP pretreatment. LC3‐II (B), p62 (C), and GAPDH expression levels from lysates were detected by western blot. A representative western blot is shown in the upper panel, with the corresponding statistical graph in the lower panel. Data are expressed as means ± SD from three independent cell cultures. For LC3‐II: ^∗^ *p* < 0.05 vs. DMSO in each group; ^#^ *p* < 0.05 vs. DMSO in vehicle or 3‐TYP groups; ^&^ *p* < 0.05 vs. DMSO in ATO + HKL group. For p62: ^∗^ *p* < 0.05 vs. vehicle; ^#^ *p* < 0.05 vs. vehicle + ATO; ^&^ *p* < 0.05 vs. ATO + HKL (two‐way ANOVA with Tukey’s post hoc test).

We assessed alterations in autophagic flux by analyzing the autophagy‐related protein LC3‐II in vehicle‐ and ATO‐treated cardiomyocytes. Western blot showed no significant change in the LC3‐II/GAPDH ratio in cardiomyocytes treated with ATO (Figure 5A). To further detect changes in autophagic flux, we incubated cardiomyocytes with bafilomycin A1 (0.1 μmol/L) in each group. As shown in Figure 5A, bafilomycin A1 administration induced a significant increase in the LC3‐II/GAPDH ratio in vehicle‐treated cardiomyocytes. However, the LC3‐II/GAPDH ratio was not elevated in ATO‐stimulated cardiomyocytes, indicating inhibited autophagic flux. In contrast, HKL pretreatment elevated LC3‐II levels in ATO‐treated cardiomyocytes to those of the sham group and further increased LC3‐II levels in ATO‐treated cardiomyocytes when bafilomycin A1 was concomitantly used to block autophagic flux. These results suggest that ATO reduces autophagic flux in cardiomyocytes, while HKL pretreatment restores the inhibited autophagic flux.

To investigate whether SIRT3 is the target through which HKL restores ATO‐induced inhibition of autophagic flux, we used 3‐TYP to inhibit SIRT3. As shown in Figure 5B, after 3‐TYP administration, the LC3‐II/GAPDH ratios in HKL + ATO cotreated cardiomyocytes were equivalent to those in ATO‐stimulated cells, indicating that SIRT3 inhibition abolished the protective effect of HKL against ATO‐induced blockage of autophagic flux. Meanwhile, p62 levels were significantly increased in cardiomyocytes treated with 4 μM ATO, which was prevented by HKL pretreatment. Likewise, 3‐TYP administration abrogated the reduction in p62 levels in HKL + ATO cotreated cardiomyocytes (Figure 5C). Overall, these data indicate that HKL restores ATO‐induced impairment of autophagic flux via SIRT3 activation.

### 3.5. Autophagic Flux Blocker Eliminates the Protective Effect of Honokiol on ATO‐Induced Cardiotoxicity in Cultured Cardiomyocytes

**FIGURE 6:**
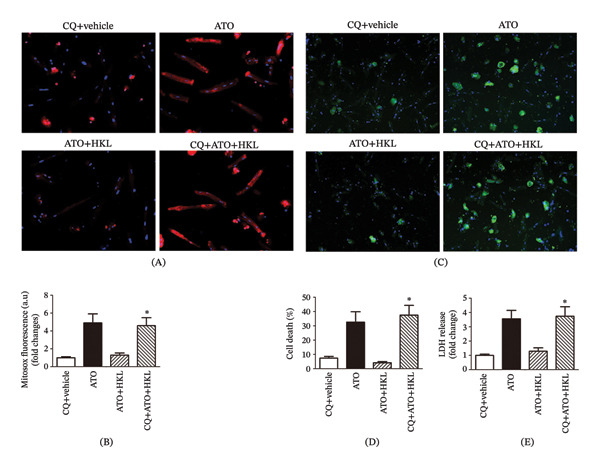
Autophagic flux blocker offsets the protective effect of Honokiol on ATO‐induced cardiotoxicity in cultured cardiomyocytes. The effect of HKL on ATO myocardial toxicity was observed with or without preincubation with chloroquine. Mitochondrial ROS production (A, B), cell death (C, D), and LDH leakage (E) were measured. Data are expressed as means ± SD from three independent cell cultures. ^∗^ *p* < 0.05 vs. ATO + HKL (two‐way ANOVA with Tukey’s post hoc test).

To explore whether HKL exerts cardioprotective effects by improving autophagic flux, cardiomyocytes were preincubated with chloroquine (CQ, a potent autophagic flux blocker) followed by HKL pretreatment in the presence of ATO. As shown in Figure 6, CQ reversed the HKL‐induced inhibition of mitochondrial ROS production (Figure 6A,B) and cardiomyocyte death (Figure 6C–E). These results demonstrate that autophagic flux is closely associated with the protective effect of HKL against ATO cardiotoxicity in cultured cardiomyocytes.

### 3.6. Autophagic Flux Blocker Offsets the Protective Effect of Honokiol on ATO‐Induced Ferroptosis in Myocardial Tissues

**FIGURE 7:**
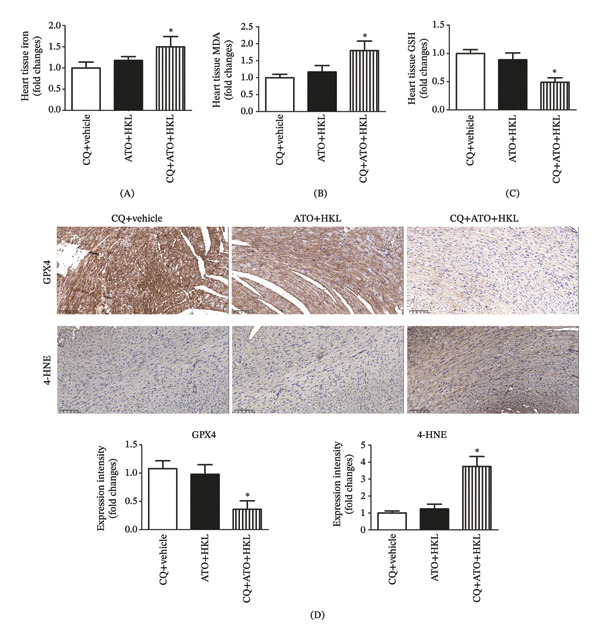
Autophagic flux blocker offsets the protective effect of Honokiol on ATO‐induced ferroptosis in myocardial tissues. Chloroquine was administered daily to ATO + HKL‐treated mice. Three days after the last administration, animals were sacrificed, and hearts were collected. Iron content (A), MDA levels (B), and GSH levels (C) of heart tissues were measured. Heart tissues were embedded in paraffin and sectioned at 4‐μm thickness. GPX4 and 4‐HNE expressions were analyzed by immunohistochemistry (magnification = 200×) (D). Data are expressed as means ± SD, *n* = 6–8. ^∗^ *p* < 0.05 vs. ATO + HKL (two‐way ANOVA with Tukey’s post hoc test).

To further investigate whether autophagic flux is involved in the protective effect of HKL against ATO‐induced ferroptosis, chloroquine was administered daily to ATO + HKL‐treated mice. After four rounds of treatment, hearts were collected, and ferroptosis‐associated markers were examined. As shown in Figure 7, blockage of autophagic flux abolished the inhibition of ATO‐induced ferroptosis by HKL, as evidenced by the increased cardiac tissue iron content and MDA levels (Figure 7A,B), increased 4‐HNE expression (Figure 7D), and decreased cardiac tissue GSH and GPX4 expressions (Figure 7C,D).

## 4. Discussion

The cardiotoxicity of ATO remains a major limitation in its clinical application. Activation of sirtuin family members has been shown to alleviate ATO‐induced oxidative stress, thereby reducing cardiomyocyte damage [23]. However, the precise role of sirtuins in this process has not been fully elucidated. Our previous work demonstrated that SIRT3 is involved in the protective role of HKL against ATO‐induced cardiotoxicity [19]. The present study further investigated the underlying mechanism by which HKL protects cardiomyocytes from ATO‐induced injury, revealing a novel SIRT3‐autophagic flux‐ferroptosis axis.

SIRT3 is a mitochondrial deacetylase that regulates numerous mitochondrial proteins, including those involved in the tricarboxylic acid cycle, forkhead box protein O3a (FOXO3a), and superoxide dismutase [24, 25]. Recent studies have shown that SIRT3 plays an important role in cardiovascular physiology and pathology and impaired SIRT3 deacetylase activity predisposes cardiac tissue to dysfunction [26]. SIRT3 regulates fatty acid oxidation, reducing accumulation of lipid peroxides and other reactive byproducts that contribute to oxidative stress [27]. SIRT3 also influences metabolic pathways that respond to nutrient availability and cellular stress, modulating pathways such as AMP‐activated protein kinase (AMPK) signaling, which helps adapt cellular metabolism to stress conditions and promotes antioxidant responses [28]. Our previous studies demonstrated the role of the SIRT3–AMPK axis in HKL‐mediated cardioprotection against ATO toxicity. Additionally, accumulating evidence indicates that SIRT3 is closely associated with the cardiac mitochondrial antioxidant enzyme system [29, 30]. One of its critical targets is superoxide dismutase 2 (SOD2), a mitochondrial enzyme that converts superoxide radicals into less harmful hydrogen peroxide. SIRT3 deacetylates and activates SOD2, thereby enhancing antioxidant capacity and reducing oxidative stress [31]. Upregulation of SIRT3 expression or activity has been shown to ameliorate oxidative stress damage caused by various pathological stimuli. For instance, resveratrol reduces intracellular ROS production by upregulating SIRT3 expression, thereby inhibiting H_2_O_2_‐induced H9c2 cell death [32]. Moreover, HKL has been shown to reduce ROS levels by activating SIRT3 against doxorubicin‐induced myocardial damage [18]. In this study, we found that HKL counteracts ATO‐induced oxidative damage in cardiomyocytes. However, HKL was unable to neutralize cardiac oxidative damage induced by ATO in SIRT3‐KO mice, suggesting that HKL exerts its antioxidant effects not through direct radical scavenging but by activating SIRT3, which in turn deacetylates and activates downstream antioxidant defense systems. Western blot and immunohistochemistry showed that HKL alleviated ATO‐induced upregulation of acetylated SOD2, while SIRT3 knockout eliminated this antioxidant effect. Mechanistically, the preservation of mitochondrial function by HKL was further supported by the maintenance of cellular ATP levels. SIRT3 knockout not only exacerbated oxidative damage but also led to a significant depletion of ATP, indicating that the deacetylase activity of SIRT3 is critical for sustaining mitochondrial electron transport chain integrity and energy production under ATO challenge.

SIRT3 maintains mitochondrial function by deacetylating and activating various mitochondrial proteins involved in energy production and ROS detoxification. Healthy mitochondria are better equipped to handle oxidative stress, thereby lowering the risk of ferroptosis. Ferroptosis was recently reported to be a critical mechanism promoting ATO‐induced cardiotoxicity [20]. Unlike previous studies using H9c2 cell lines, we demonstrated that the ferroptosis inhibitor Fer‐1, administered for 12 h before ATO exposure, suppressed ATO‐induced death of primary cultured cardiomyocytes. Furthermore, ferroptosis‐associated markers were significantly induced in hearts of ATO‐treated mice, accompanied by marked downregulation of SIRT3 expression. HKL pretreatment attenuated iron overload and reversed ATO‐induced increases in MDA and 4‐HNE, as well as decreases in GSH and GPX4, in a SIRT3‐dependent manner. One of the primary ways SIRT3 influences ferroptosis is through its effect on GPX4, an essential enzyme in protecting cells from lipid peroxidation. SIRT3 promotes GPX4 expression and activity by deacetylating and activating it [33]. GPX4 reduces lipid peroxides to nontoxic lipid alcohols, thus preventing accumulation of lipid peroxides that drive ferroptosis. SIRT3 also influences iron metabolism, which is crucial for ferroptosis since iron catalyzes ROS and lipid peroxide formation. By regulating mitochondrial iron levels and iron‐related proteins, SIRT3 modulates the cellular iron pool and its availability, thereby impacting ferroptosis [34]. Our previous study demonstrated that HKL effectively attenuates ATO‐induced cardiomyocyte death, including reversal of ATO‐induced downregulation of myocardial SIRT3 expression. Therefore, SIRT3 may be a direct target through which HKL prevents cardiomyocyte ferroptosis. The present study shows that SIRT3 knockout attenuated the protective effects of HKL against ATO‐induced cardiac ferroptosis, indicating that HKL inhibits myocardial oxidative stress injury and ferroptosis by activating the SIRT3 signaling pathway. While Honokiol has been previously shown to inhibit apoptosis in various cardiac injury models [17–19], the present study reveals an additional mechanism: suppression of ferroptosis via SIRT3 activation. These two forms of regulated cell death may overlap in ATO‐induced cardiotoxicity, and Honokiol appears to modulate both through distinct but interconnected pathways.

Arsenic trioxide is a protoplasmic toxin with high affinity for protein sulfhydryl groups. Invading arsenic can combine with two sulfhydryl or hydroxyl groups on protein molecules to form stable complexes or cyclic compounds, thus destroying protein structure and function [35]. Autophagy is a process of degradation, recycling, and reuse of intracellular wastes (such as damaged organelles and proteins). Stable and unobstructed autophagic flux is essential for maintaining intracellular homeostasis. Impaired autophagy has been confirmed as an important cause of ATO myocardial toxicity [6]. Dysregulated autophagic flux influences intracellular iron availability and lipid metabolism, which are central to ferroptosis [36]. Changes in p62 and LC3‐II protein levels can reflect changes in autophagic flux; however, LC3‐II expression at a single time point does not fully reflect autophagy dynamics. Drugs that block lysosomal degradation (such as chloroquine and bafilomycin A1) should be used to determine the degree of autophagy under intervention [37]. In this study, our in vitro experiments showed that LC3‐II expression did not change significantly under ATO treatment, and there was no obvious accumulation after concomitant use of bafilomycin A1. ATO significantly accumulated p62 protein levels in primary cultured cardiomyocytes, suggesting that ATO leads to inhibition of autophagic flux. In contrast, HKL pretreatment restored the inhibited autophagic flux. To further confirm the role of autophagic flux improvement in HKL‐mediated cardioprotection, we used CQ to intervene in autophagic flux. CQ is a classical autophagic flux blocker that inhibits autophagosome–lysosome fusion [38]. When autophagic flux was blocked by CQ, the protective effect of HKL on cardiomyocytes was abrogated, as measured by oxidative stress and cell death. Meanwhile, blockage of autophagic flux also eliminated the inhibitory effect of HKL on ferroptosis, indicating that HKL alleviates ATO‐induced myocardial toxicity by improving autophagic flux.

SIRT3 exerts its role in regulating autophagy by modifying various proteins through deacetylation. For instance, SIRT3 deacetylates and activates Forkhead box O3 (FoxO3), a transcription factor that promotes expression of autophagy‐related genes. SIRT3 involvement in regulating autophagy helps clear damaged organelles and proteins, including those involved in iron and lipid metabolism. Effective autophagy reduces cellular stress and prevents conditions that promote ferroptosis [36]. Recently, SIRT3 has been shown to affect cardiomyocyte autophagy by activating certain signaling pathways. It modulates AMPK and mammalian target of rapamycin (mTOR), two key autophagy regulators. SIRT3 activates AMPK, which stimulates autophagy, while inhibiting mTOR, a negative regulator of autophagy. This interplay between SIRT3, AMPK, and mTOR helps fine‐tune the autophagic response in cardiomyocytes [39]. While HKL has been previously shown to inhibit apoptosis in various cardiac injury models, the present study reveals an additional mechanism: suppression of ferroptosis via SIRT3 activation. These two forms of regulated cell death may overlap in ATO‐induced cardiotoxicity, and HKL appears to modulate both through distinct but interconnected pathways.

In recent years, with deepening understanding of autophagy’s role in heart disease pathogenesis and progression, targeting autophagy has emerged as a potential therapeutic strategy. Natural products with autophagy‐modulating abilities have become potential candidates for heart disease treatment due to their wide safety margin and low toxicity. HKL has been shown to induce autophagy in cardiomyocytes, associated with cardioprotective effects. Autophagy activation by HKL protects against various forms of cardiac injury, including ischemia–reperfusion injury [21] and anti‐β1‐adrenergic receptor autoantibody‐induced cardiotoxicity [22]. By enhancing clearance of damaged components and maintaining cellular homeostasis, HKL‐mediated autophagy contributes to cardiomyocyte survival and functional recovery. In our previous studies, HKL effectively alleviated ATO‐induced myocardial hypertrophy and dysfunction. In this pathological and pharmacological process, ATO‐induced downregulation of myocardial AMPK phosphorylation and SIRT3 expression was reversed by HKL preconditioning. To explore the role of SIRT3 in HKL‐rectified abnormal myocardial autophagic flux, we used 3‐TYP to inhibit SIRT3 activity. SIRT3 inhibition abolished the effects of HKL on both autophagic flux restoration and ferroptosis markers, indicating that HKL exerts its cardioprotective effects against ATO primarily through the SIRT3/AMPK signaling pathway. This SIRT3‐dependent protection involves interference with multiple steps of the ferroptotic cascade. ATO induces ferroptosis by promoting iron overload, lipid peroxidation, and GPX4 inactivation. HKL appears to intervene at multiple stages: it reduces mitochondrial ROS, restores GPX4 expression, and enhances autophagic clearance of damaged organelles, thereby limiting ferroptosis progression.

In conclusion, the present study confirms that HKL inhibits oxidative stress injury and ferroptosis in cardiomyocytes by activating the SIRT3 signaling pathway, thereby alleviating ATO cardiotoxicity. Additionally, this study demonstrates for the first time that ATO causes inhibition of autophagic flux in cardiomyocytes, and HKL promotes autophagic flux through SIRT3 activation, which is a key factor in combating ATO myocardial toxicity.

**Nomenclature**
ATO: Arsenic trioxide
HKL: Honokiol
Fer‐1: Ferrostatin‐1
ROS: reactive oxygen species
GPX4: glutathione peroxidase 4
MDA: malondialdehyde
GSH: glutathione
WGA: wheat germ agglutinin
4‐HNE: 4‐Hydroxynonenal
SIRT3: sirtuin 3
SOD2: superoxide dismutase 2
CQ: chloroquine
3‐TYP: SIRT3 inhibitor
KO: knockout
WT: wild‐type
EF: ejection fraction
FS: fractional shortening
ATP: adenosine triphosphate.

## Author Contributions

Qing‐qing Wei and Fan Yang designed and conducted the experiments and wrote the paper. Qing‐qing Wei, An‐liang Huang, and Ning‐yi Liu conducted the experiments. Qing‐qing Wei and Yi‐ran Zhang performed data analysis. An‐liang Huang and Fan Yang designed the experiments and supervised the research. All authors discussed the results and commented on the manuscript.

## Funding

This study was supported by grants from the Chengdu Medical Research Project of Chengdu Health Commission, China (No. 2023164), and the Technological Innovation Research and Development Projects of Chengdu Science and Technology Bureau, China (No. 2022‐YF05‐01725‐SN).

## Disclosure

A preprint of this work was previously published on Research Square [40].

## Ethics Statement

Ethical approval was obtained from the Ethics Committee of State Key Laboratory of Biotherapy, Sichuan University (approval no: 20230302090).

## Conflicts of Interest

The authors declare no conflicts of interest.

## Supporting Information

Additional supporting information can be found online in the Supporting Information section.

## Supporting information


**Supporting Information** Honokiol preserves cardiac ATP levels in a SIRT3‐dependent manner following ATO exposure. (A) ATP production capacity in freshly isolated cardiac mitochondria from the indicated groups (*n* = 4 per group). (B) Total ATP content in heart tissue lysates (*n* = 6 per group). Data are expressed as means ± SD. ^∗^ *p* < 0.05 vs. vehicle; ^#^ *p* < 0.05 vs. ATO; ^&^ *p* < 0.05 vs. ATO + HKL (two‐way ANOVA with Tukey’s post hoc test).

## Data Availability

The data supporting the findings of this study are available from the corresponding author upon reasonable request.
