# Supplementary material for: Honokiol Inhibits Arsenic Trioxide–Induced Cardiomyopathy by Modulating Ferroptosis via SIRT3 Signaling Pathway
Source: Cardiol Res Pract. 2026 Aug 2;2026:8878178. doi: 10.1155/crp/8878178 (PMC13430285; doi:10.1155/crp/8878178)
Supplement: Supplementary file 1 — Supporting Information Honokiol preserves cardiac ATP levels in a SIRT3‐dependent manner following ATO exposure. (A) ATP production capacity in freshly isolated cardiac mitochondria from the indicated groups (n = 4 per group). (B) Total ATP content in heart tissue lysates (n = 6 per group). Data are expressed as means ± SD. ∗ p < 0.05 vs. vehicle; # p < 0.05 vs. ATO; & p < 0.05 vs. ATO + HKL (two‐way ANOVA with Tukey’s post hoc test). [file CRP-2026-8878178-s001.docx]

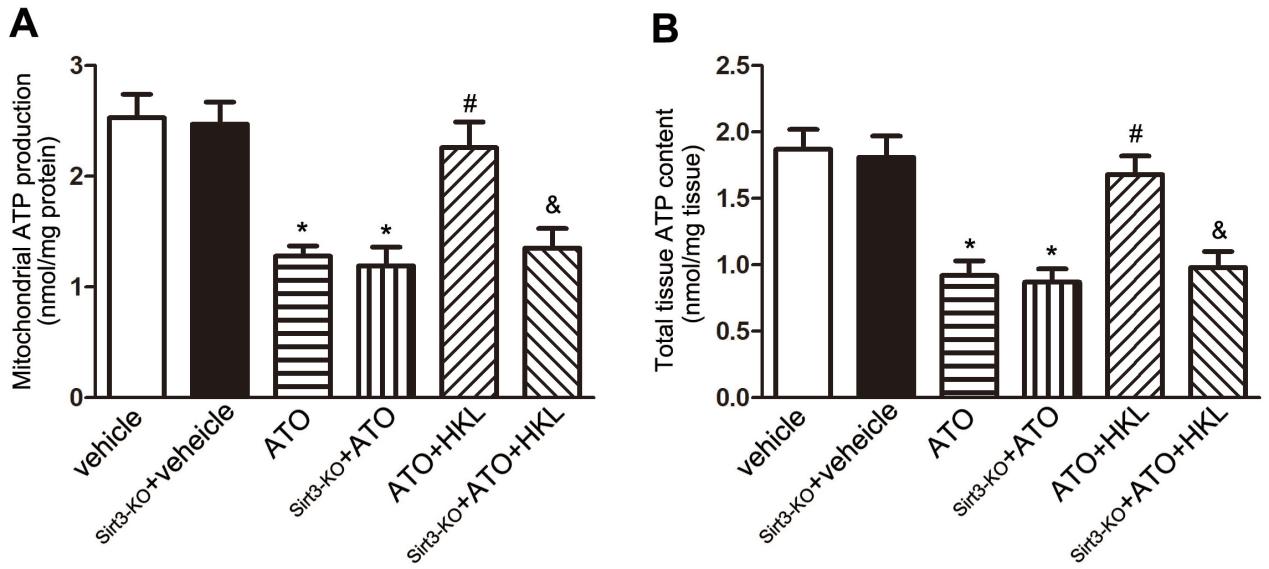


Supplemental Figure 1: Honokiol preserves cardiac ATP levels in a SIRT3-dependent manner following ATO exposure. (A) ATP production capacity in freshly isolated cardiac mitochondria from the indicated groups (n=4 per group). (B) Total ATP content in heart tissue lysates (n=6 per group). Data are expressed as means ± SD. *p < 0.05 vs. vehicle; #p < 0.05 vs. ATO; &p < 0.05 vs. ATO+HKL (two-way ANOVA with Tukey's post-hoc test).
